# Increase of PRPP enhances chemosensitivity of PRPS1 mutant acute lymphoblastic leukemia cells to 5‐Fluorouracil

**DOI:** 10.1111/jcmm.13907

**Published:** 2018-09-25

**Authors:** Dan Wang, Yao Chen, Houshun Fang, Liang Zheng, Ying Li, Fan Yang, Yan Xu, Lijuan Du, Bin‐Bing S. Zhou, Hui Li

**Affiliations:** ^1^ Key Laboratory of Pediatric Hematology and Oncology Ministry of Health Shanghai Children's Medical Center Shanghai Jiao Tong University School of Medicine Shanghai China; ^2^ Pediatric Translational Medicine Institute Shanghai Children's Medical Center Shanghai Jiao Tong University School of Medicine Shanghai China; ^3^ Department of Pharmacology and Chemical Biology School of Basic Medicine and Collaborative Innovation Center for Translational Medicine Shanghai Jiao Tong University School of Medicine Shanghai China; ^4^ Department of Integrative Oncology Fudan University Shanghai Cancer Center Shanghai China; ^5^ Department of Emergency Qingdao Municipal Hospital Shandong China

**Keywords:** 5‐FU, acute lymphoblastic leukemia, nucleotide metabolism, PRPP, PRPS1

## Abstract

Relapse‐specific mutations in *phosphoribosyl pyrophosphate synthetase 1* (*PRPS1*), a rate‐limiting purine biosynthesis enzyme, confer significant drug resistances to combination chemotherapy in acute lymphoblastic leukemia (ALL). It is of particular interest to identify drugs to overcome these resistances. In this study, we found that PRPS1 mutant ALL cells specifically showed more chemosensitivity to 5‐Fluorouracil (5‐FU) than control cells, attributed to increased apoptosis of PRPS1 mutant cells by 5‐FU. Mechanistically, PRPS1 mutants increase the level of intracellular phosphoribosyl pyrophosphate (PRPP), which causes the apt conversion of 5‐FU to FUMP and FUTP in Reh cells, to promote 5‐FU‐induced DNA damage and apoptosis. Our study not only provides mechanistic rationale for re‐targeting drug resistant cells in ALL, but also implicates that ALL patients who harbor relapse‐specific mutations of PRPS1 might benefit from 5‐FU‐based chemotherapy in clinical settings.

## 1 INTRODUCTION

Acute lymphoblastic leukemia (ALL) is the most common childhood cancer, accounting for more than 25% of all childhood cancers.^1^ Although risk‐stratified combination chemotherapy has improved the cure rate of ALL patients, relapsed ALL remains a leading cause of mortality among all childhood malignancies.^2^ The thiopurines 6‐thioguanine (6‐TG) and 6‐mercaptopurine (6‐MP) are key drugs in maintenance therapy which will continue for the next two to two‐and‐a‐half years after initial remission, and both are prodrugs converted by the purine salvage pathway to cytotoxic thioguanine nucleotides.^3^ Therefore, dysfunction of the enzymes involved in purine metabolism has been associated with the ALL recurrence.^4^ We previously identified multiple relapse‐specific mutations in *phosphoribosyl pyrophosphate synthetase 1 gene* (*PRPS1*), which encodes a rate‐limiting purine biosynthesis enzyme, in 24/358 (6.7%) relapsed childhood B‐ALL samples. Among these mutations, the encoding residues A190 and S103 are hotspot mutation sites presented in 11/24 and 4/24 patients, respectively. Mechanistically, negative feedback‐defective PRPS1 mutants result in constitutive activation of purine biosynthesis and accumulation of purine nucleotides in cells. Increased hypoxanthine (HX) competitively inhibits activation of 6‐MP and 6‐TG, leading to thiopurine resistance and tumor relapse.^5^ In addition to 6‐MP and 6‐TG, PRPS1 mutant cells also confer slight resistance to other commonly used ALL chemotherapeutic drugs, such as methotrexate (MTX) and cytosine arabinoside (Ara‐C). However, no drug has been shown to overcome relapse‐specific PRPS1 mutant‐conferred drug resistance and to directly target PRPS1 mutant cells.

5‐Fluorouracil (5‐FU) as an antimetabolite drug is widely used in the treatment of a range of cancers.^6^, ^7^ 5‐FU is converted intracellularly to three main active metabolites: fluorodeoxyuridine monophosphate (FdUMP), fluorodeoxyuridine triphosphate (FdUTP) and fluorouridine triphosphate (FUTP), these active metabolites can disrupt the synthesis of DNA/RNA and inhibit thymidylate synthase (TS).^8^, ^9^ The main mechanism of 5‐FU activation features its conversion to FUMP, either directly by orotate phosphoribosyltransferase (OPRT) with phosphoribosyl pyrophosphate (PRPP) as a cofactor, or indirectly via fluorouridine (FUR) through the sequential action of uridine phosphorylase (UP) and uridine kinase (UK). Notably, PRPP, as the catalytic product of PRRS1, is also the precursor of *de novo* pyrimidine biosynthesis. Dihydropyrimidine dehydrogenase (DPD)‐mediated conversion of 5‐FU to dihydrofluorouracil (DHFU) is the rate‐limiting step of 5‐FU catabolism in normal and tumor cells, and up to 80% of administered 5‐FU is broken down by DPD in the liver.^9^ Numerous studies have shown that expression levels of TS, DPD and OPRT could affect tumor cell sensitivity to 5‐FU.^10^, ^11^, ^12^, ^13^, ^14^ However, it remains unknown whether 5‐FU has effective role in treating ALL, and especially targeting PRPS1 mutant ALL cells.

In this study, we demonstrated that 5‐FU can specifically sensitize ALL cells with PRPS1 mutants (A109T and S103T) by increasing DNA damage and apoptosis. We also found that 5‐FU is more inclined to be converted to FUMP and FUTP instead of FdUMP in PRPS1 mutant cells. Mechanistically, accumulated intracellular PRPP promotes 5‐FU prodrug activation and confers enhanced sensitivity to 5‐FU on PRPS1 mutant cells. Our findings would bridge between PRPS1 mutant‐induced metabolic abnormality and prodrug activation of 5‐FU, suggesting a potential therapeutic strategy for drug resistant ALL patients with relapse‐specific *PRPS1* mutations.

## 2 MATERIALS AND METHODS

### 2.1 Reagents

Fetal bovine serum (FBS) and RPIM‐1640 medium (Gibco, Grand Island, NY, USA); 5‐FU, 6‐MP, 6‐TG, DXR, VCR, HU, cisplatin and PRPP (Sigma‐Aldrich, St. Louis, USA); Annexin V‐APC/PI Apoptosis Detection Kit (MultiSciences, Hangzhou, China); FuGENE‐6 and CellTiter‐Glo Luminescent kit (Promega, Madison, WI, USA); Amicon purification column‐100kD (Millipore, Burlington, MA, USA); tissue culture plate (Corning, NY, USA); IRDye800COR‐lgG second antibody (LI‐COR, Lincoln, Nebraska, USA); nitrocellulose membrane 0.45 μm (GE Healthcare, Chicago, IL, USA); [U‐^13^C_6_]‐d‐glucose (Cambridge Isotope Laboratories, Andover, MA, USA, cat. No. CLM‐1396‐1).

### 2.2 Cell culture

The human lymphoblastic leukemia Reh, Jurkat and Nalm‐6 cells were cultured in RPMI‐1640 medium supplemented with 10% FBS, 100 U/mL penicillin G and 100 μg/mL streptomycin. HCT116 cells were cultured in McCoy's 5a Medium supplemented with 10% FBS, 100 U/mL penicillin G and 100 μg/mL streptomycin. SW480 and HEK293T cells were cultured in Dulbecco's Modified Eagle Medium supplemented with 10% FBS, 100 U/mL penicillin G and 100 μg/mL streptomycin. All cells were incubated at 37°C under 5% CO_2_. Cell lines were regularly authenticated and tested for mycoplasma contamination.

### 2.3 Lentivirus production and infection

Lentivirus expression plasmids of wild‐type and mutant PRPS1 were described in our previous report.^5^ Lentivirus production was performed as described previously.^5^ Briefly, lentiviral constructs were packaged in plasmids PSPAX2 and PMD2G and transfected into HEK293T cells using FuGENE‐6 to produce viruses. Supernatant was used to infect Reh cells after concentration. GFP‐positive cells were sorted in a MofloXDP.

### 2.4 Metabolites extraction and LC‐MS

Cells were plated in 6‐well plates at 2 × 10^6^ cells per well in standard medium. For 5‐FU metabolites measurement, cells were cultured in medium containing 10 μg/mL 5‐FU for 24 hours. For PRPP measurement, cells were cultured in RPMI 1640 and incubated with [U‐^13^C_6_]‐d‐glucose for 5 minutes. At the end of incubations, cells were rapidly washed two times with cold PBS and extracted with ice‐cold extraction solution composed of 80% Methanol in water (1000 μL/2 × 10^6^ cells). The lysates were vortexed for 10 minutes at 4°C and immediately centrifuged at 15 000 rpm for 15 minutes at 4°C. The supernatants were collected and analyzed by LC‐MS.

For the LC separation, a ZIC‐pHILIC (150 × 2.1 mm, SeQuant, Darmstadt, Germany) with a guard column (20 × 2.1 mm, SeQuant, Darmstadt, Germany) was used. The mobile phase A was 20 mmol/L ammonium carbonate plus 0.1% ammonia hydroxide in water and mobile phase B was acetonitrile. The flow rate was 200 μL/mL and gradient as follows: 0 minutes 80% of B to 25 minutes 20% of B and the column was then re‐equilibrated until 32 minutes at 80% of B. The Exactive Plus Orbitrap mass spectrometer (Thermo Scientific, Carlsbad, CA, USA) was operated in a polarity switching mode.

### 2.5 Cell viability assay

Cell viability and IC50 was determined as described previously.^5^ Briefly, cells were plated in 96‐well plates (12 000 cells per well) and treated for 72 hours with serially diluted drugs. Cell viability was determined using CellTiterGlo Luminescent kit according to manufacturer's instructions. IC50 value was calculated through GraphPad Prism software. All experiments were performed in triplicate, and results were calculated as mean ± SD.

### 2.6 Cell survival

Cells were plated in 96‐well plates (15 000 cells per well) with three replicates. Cell viability was measured through CellTiter‐Glo reagents at different time points (0, 24, 48 and 72 hours) after dosing. Relative proliferation rate at different time points was calculated with 0 hour as control.

### 2.7 Annexin V/propidium iodide (PI) analysis

Apoptosis analyzed by Annexin V/PI was described previously.^5^ Briefly, cells were plated in 24‐well plates (1 × 10^6^ cells/1 mL medium) and treated with 1.5 μg/mL 5‐FU for 72 hours. Apoptosis was measured by staining with annexin V‐APC and PI followed by flow cytometry.

### 2.8 Western blot analysis

Western blot was performed as described previously.^5^ Briefly, cells were plated in 12‐well plates (1.5 × 10^6^ cells/1.5 mL medium) and treated with 1.5 μg/mL 5‐FU for 48 hours. Cells were harvested in 1× sodium dodecyl sulfate (SDS) lysis buffer and analyzed via SDS‐polyacrylamide gel electrophoresis with the following antibodies: H2AX‐S139 (Abcam, Cambridge, UK, 1:3000 dilution), H2AX (Abcam, 1:3000 dilution), PARP (Abcam, 1:1000 dilution), cleaved PARP (Abcam, 1:1000 dilution), TS (Abcam, 1:1000 dilution), TK (Abcam, 1:1000 dilution), OPRT (Abcam, 1:1000 dilution), His‐tag (HuaBio, Hangzhou, China, 1:1000 dilution), Actin (HuaAn Biotechnology, 1:5000 dilution), and GAPDH (HuaAn Biotechnology, 1:5000 dilution). Immunoblots were analyzed using the Odyssey system.

### 2.9 Gene knockdown

Gene knockdown was performed as described previously.^5^ Briefly, clustered regularly interspaced short palindromic repeats (CRISPR)/Cas9 system was used to knock out genes in Reh cells accordingly. CRISPRs were designed based on information available at http://crispr.mit.edu and cloned into lentiCRISPR/Cas9 vector by following the Zhang laboratory's protocol. The sequence targeted by PRPS1, TK and OPRT CRISPR are 5′‐GAAATTGGTGAAAGTGTACG‐3′; 5′‐CACCGGAACAGATAACTGTAGCCAA‐3′; 5′‐CACCGCAAGACCCGGGGGCAGATCC‐3′, respectively. The cells were infected with lentivirus and selected by using puromycin. Mixed cells were subjected to further experiments.

### 2.10 Mouse Models and Chemotherapy

Xenograft models of human ALL were established in B‐NDG (NOD‐*Prkdc* ^*scid*^ *IL2rg* ^*tm1*^/Bcgen) mice (Biocytogen). GFP‐labeled Reh PRPS1 WT and mutant cell transplantations were performed by tail vein injection (1 × 10^7^ cells for each) in mice aged 5 weeks. After transplantation, the mice were monitored to detect the appearance of leukemic symptoms, such as weight loss, hunch‐back and decreased activity. Then leukemic mice were treated with 5‐FU (100 mg/kg, intraperitoneally) for 3 days. After chemotherapy, the mice were sacrificed for isolating bone marrow cells from tibias. GFP^+^ cells were analyzed by flow cytometry.

### 2.11 Statistical analysis

All data were analyzed by GraphPad Prism 6.0 software. Comparison between two groups was performed using *t* test. Data for survival curve were analyzed via two‐way ANOVA (multiple comparison‐analysis of different treatment groups at the same time). Data were shown as means ± SD.

## 3 RESULTS

### 3.1 5‐FU sensitizes PRPS1 mutant ALL cells

**Figure 1:**
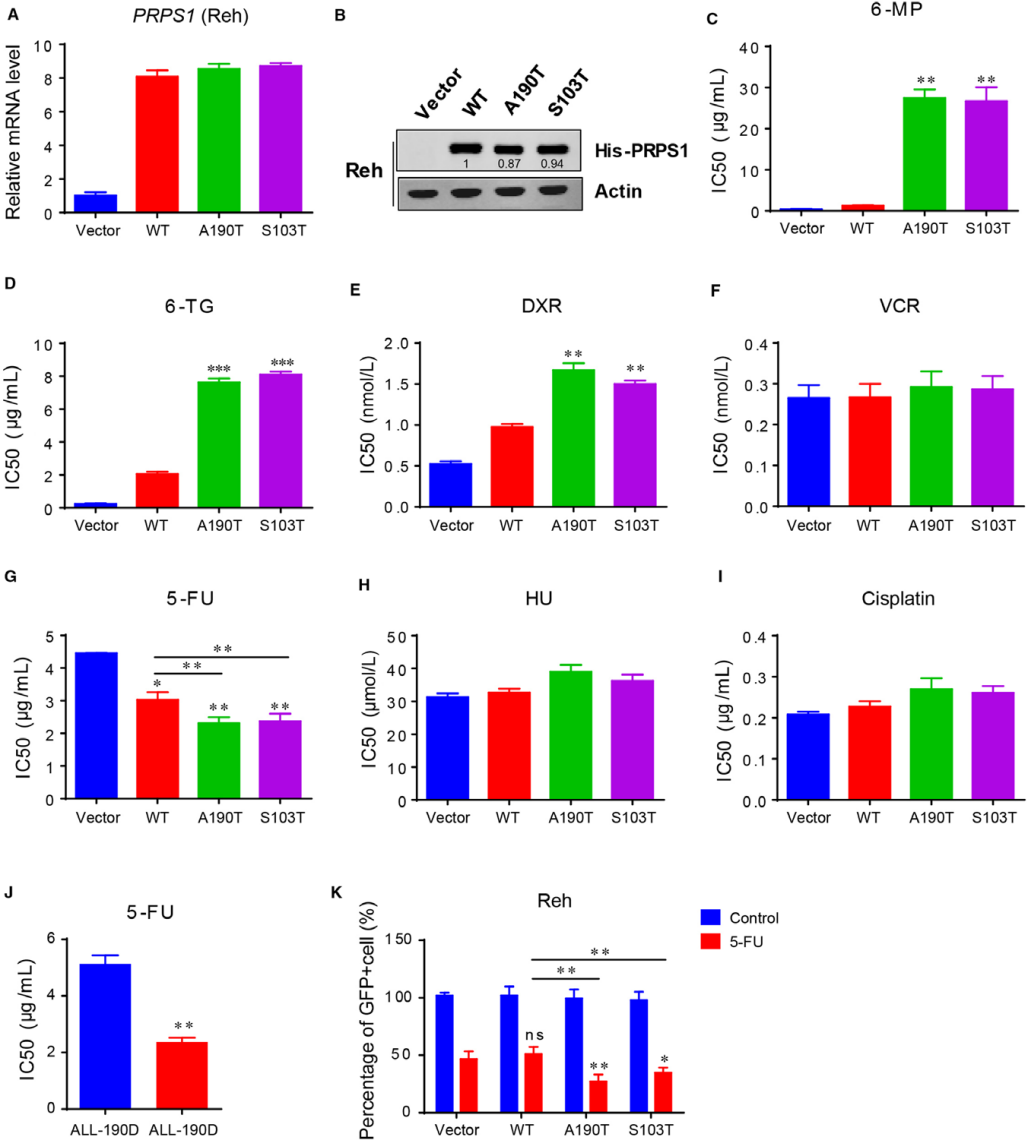
5‐FU sensitizes PRPS1 mutant ALL cells. (A and B) Stable expression of exogenous PRPS1 wild type or PRPS1 mutants (A190T and S103T) in Reh cells. The relative gray scale determination was analyzed using ImageJ software. (C–F) Screening of chemosensitivity of Reh cells (PRPS1 WT or mutants) to the chemotherapeutic drugs commonly used in ALL treatment by using cell viability assay measuring half maximal inhibitory concentration (IC50). **P < 0.01, and ***P < 0.001, two‐tailed Student's *t* tests. 6‐MP, 6‐mecaptopurine; 6‐TG, 6‐thioguanine; DXR, doxorubicin; VCR, vincristine. (G) Reh cells harboring PRPS1 mutants showed much more chemosensitivity to 5‐fluorouracil. *P < 0.05, **P < 0.01, two‐tailed Student's *t* tests. (H and I) Both of hydroxyurea (HU) and cisplatin showed little effect on the viability of Reh cells (IC50). (J) The primary ALL cells harboring PRPS1 mutant showed more chemosensitivity to 5‐FU. **P < 0.01, two‐tailed Student's *t* tests. (K) Percentage of GFP‐positive cells were dramatically reduced by 5‐FU in vivo. *P < 0.05, **P < 0.01, two‐tailed Student's *t* tests

To screen chemotherapeutic drugs which can overcome PRPS1 mutant‐driven drug resistance, we performed cell viability assay by measuring half maximal inhibitory concentration (IC50) to analyze the effects of PRPS1 wild‐type (WT) and PRPS1 mutants (A190T and S103T) in response to multiple chemotherapeutic drugs in Reh human ALL cell line. As shown in Figure 1A‐D, the viability of Reh cells harboring PRPS1 mutants dramatically increased after treatment with both 6‐MP and 6‐TG, whereas the cells harboring PRPS1 WT showed slight resistance to 6‐MP and 6‐TG, consistent with our previous findings that relapse‐specific PRPS1 mutations confer thiopurine resistance.^5^ Meanwhile, these cells had a minor resistance to doxorubicin (DXR) but no significant sensitivity to vincristine (VCR) (Figure 1E and F), both of drugs are also commonly used in ALL treatment. In addition, we also assessed the chemosensitivity of these cells to common chemotherapeutic agents for solid tumors, such as 5‐FU, hydroxyurea (HU) and cisplatin. Intriguingly, Reh cells harboring PRPS1 mutants were much more sensitive to 5‐FU, unlike PRPS1 WT cells with a moderate chemosensitivity to 5‐FU compared to the control cells (Figure 1G), despite that none of these cells showed significant chemosensitivity to HU and cisplatin compared to the control cells (Figure 1H and I). Similarly, we also found PRPS1 mutants showed more chemosensitivity to 5‐FU than PRPS1 WT did in other two ALL cell lines, Jurkat and Nalm6 (Figure 1A‐D). Furthermore, we obtained the primary ALL cells from the patients to confirm this effect. As shown in Figure 1J, the relapse cells (ALL‐190R), which harbored PRPS1 S103R mutation, were more sensitive to 5‐FU action than diagnosis samples (ALL‐190D). Additionally, the results from xenograft mice models also demonstrated that 5‐FU had better chemotherapeutic effect in vivo against PRPS1 mutant cells rather than PRPS1 WT cells (Figure 1K). Taken together, these results indicated that 5‐FU could sensitize PRPS1 mutant ALL cells.

### 3.2 5‐FU promotes the apoptosis of PRPS1 mutant ALL cells

**Figure 2:**
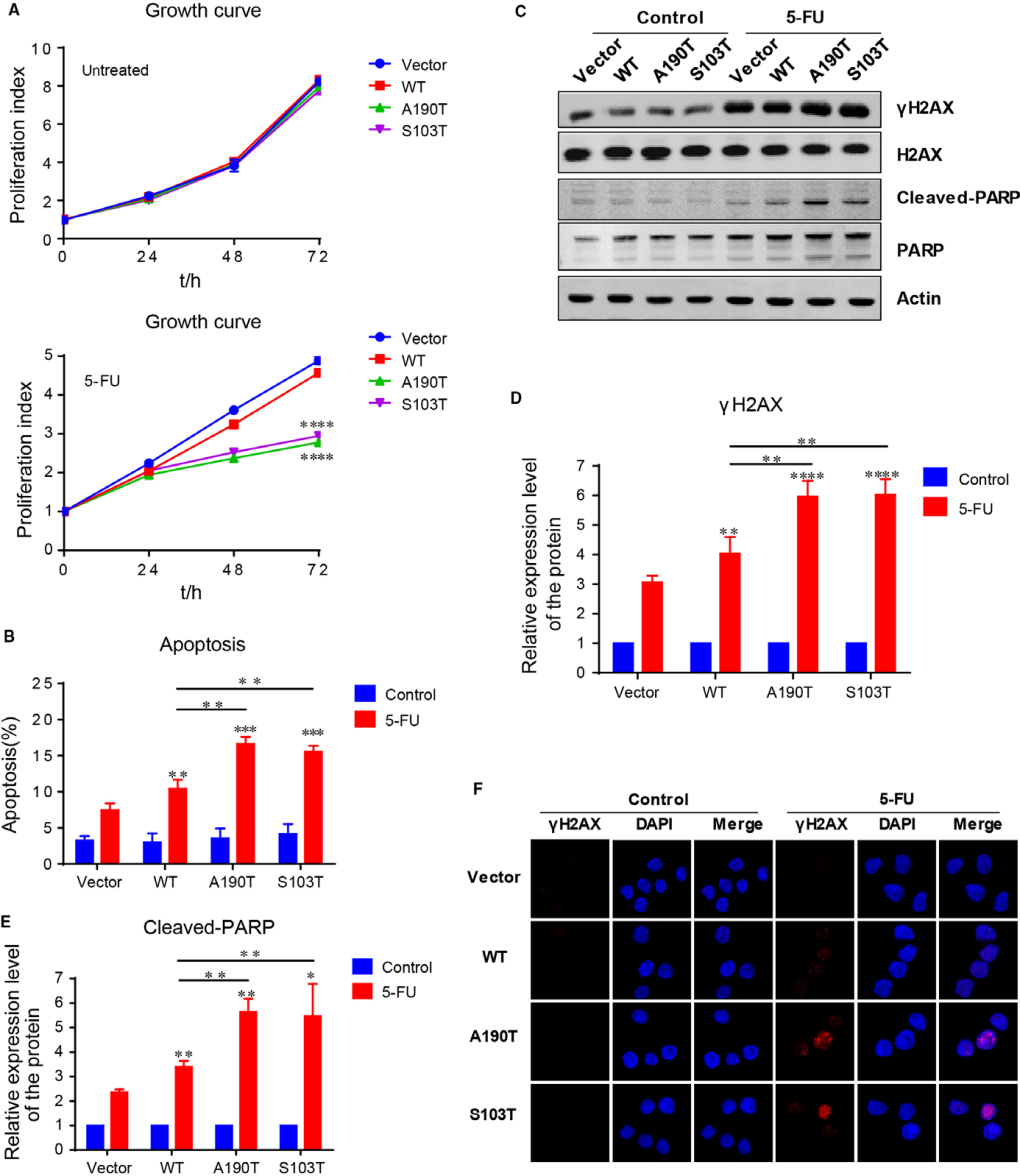
5‐FU accelerates apoptosis of PRPS1 mutant Reh cells. (A) PRPS1 mutant cells proliferated more slowly than PRPS1 WT and control cells. Cell viability was measured at 0, 24, 48, and 72 hours after adding 1.5 μg/mL 5‐FU. ****P < 0.0001, two‐way ANOVA (simultaneous multiple comparison‐analysis of different treatment groups). (B) Apoptosis induced by 5‐FU was markedly increased in PRPS1 mutant cells. Percentage of apoptotic cells was detected at 72 hours after treatment with 1.5 μg/mL 5‐FU by annexin‐V/PI staining assay. ***P < 0.001, two‐tailed Student's *t* tests. (C‐E) 5‐FU caused enhancement of DNA damage response and apoptosis in PRPS1 mutant cells compared to that in PRPS1 WT and control cells. Cells were harvested at 48 hours after treatment with 1.5 μg/mL 5‐FU and the sample wasanalyzed by Western blot. Quantification of protein level was conducted by using ImageJ software. *P < 0.05, **P < 0.01, and ****P < 0.0001, two‐tailed Student's *t* tests. (F) DDR marker yH2AX was upregulated in PRPS1 mutant cells as detected byimmunofluorescence assay at 48 hours after treatment with 1.5 μg/mL 5‐FU

We further showed that the proliferation rate of PRPS1 mutant ALL cells was almost the same as the PRPS1 WT and control ALL cells under normal circumstances (Figure 2A, Figure S1E and F). However, with the treatment of 5‐FU, the PRPS1 mutant cells proliferated more slowly than PRPS1 WT and control cells (Figure 2A, Figure S1E and F), suggesting the susceptibility of PRPS1 mutants to 5‐FU action. It has been shown that the cytotoxicity of 5‐FU is ascribed to the misincorporation of fluoronucleotides into RNA and DNA, leading to DNA damage response (DDR) and apoptosis.^15^, ^16^ We therefore reasoned that PRPS1 mutant could allow enhanced apoptosis for Reh ALL cells with the treatment of 5‐FU. As shown in Figure 2B, 5‐FU can readily induce apoptosis of Reh cells as speculated, and the cells harboring PRPS1 WT showed moderately increased apoptosis compared to the control cells. Moreover, the rate of apoptosis induced by 5‐FU in cells harboring PRPS1 mutant was much higher than that in control cells (Figure 2B). We further examined the expression of γ‐H2AX and the cleavage of PARP in these cells, which can be taken as a biomarker of DDR and a symptom of cell apoptosis, respectively. Similarly, 5‐FU treatment significantly caused DDR and apoptosis of Reh ALL cells, and also, PRPS1 mutant cells showed an enhancement of DDR and apoptosis induced by 5‐FU compared to PRPS1 WT cells (Figure 2C‐F). Taken together, these results indicated that 5‐FU restrained the proliferation of PRPS1 mutant ALL cells by promoting DNA damage response and apoptosis.

### 3.3 Apt conversion of 5‐FU to FUMP and FUTP in PRPS1 mutant ALL cells

**Figure 3:**
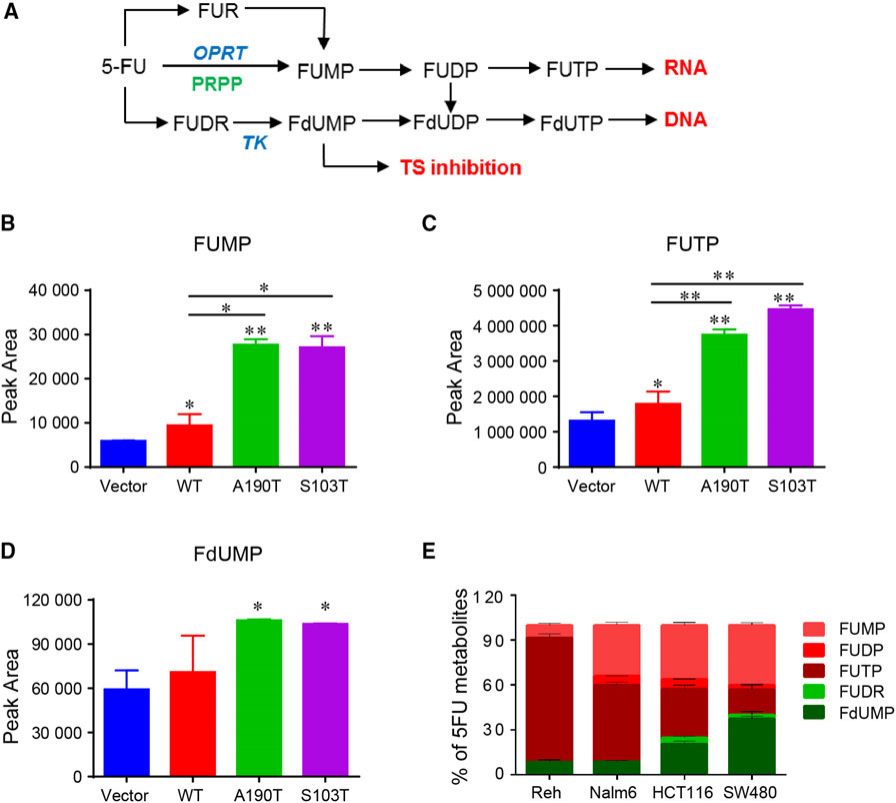
Apt conversion of 5‐FU to FUMP and FUTP in PRPS1 mutant ALL cells. (A) diagrammatic sketch of 5‐FU catabolism. OPRT, orotate phosphoribosyltransferase; TK, thymidylate kinase; TS, thymidylate synthase. (B–D) The outputs of FUMP and FUTP but not FdUMP in PRPS1 mutant cells were much higher than that in PRPS1 WT and control cells. Reh cells were harvested at 24 hours after treatment with 10 μg/mL 5‐FU. *P < 0.05, **P < 0.01, two‐tailed Student's *t* tests. (E) The ratio of 5‐FU metabolites in Reh, Nalm6, HCT116 and SW480. All cells were harvested at 24 hours after treatment with 10 μg/mL5‐FU

Eventually, 5‐FU is converted intracellularly to three main active metabolites: FUTP, which can be incorporated into RNA; FdUTP, which can be incorporated into DNA; FdUMP, which can inhibit the activity of thymidylate synthase (Figure 3A). To determine how 5‐FU sensitizes PRPS1 mutant ALL cells, we first analyzed 5‐FU metabolism in Reh cells by liquid chromatography‐mass spectrometry (LC‐MS). As shown in Figure 3B and C, although the Reh cells harboring PRPS1 WT had a slight increase in FUMP and FUTP compared to the control cells, the FUMP and FUTP in Reh cells harboring PRPS1 mutants was significantly higher than that in the cells harboring PRPS1 WT. As a note, we have not detected enough FdUTP to directly evaluate the conversion efficiency of 5‐FU to FdUTP within these cells, but we still found the conversion of 5‐FU to FdUMP, which could be further converted to FdUTP (Figure 3A). In comparison, we only observed a moderate increase of FdUMP in PRPS1 mutant cells than that in PRPS1 WT cells (Figure 3D). These results indicated that 5‐FU is more inclined to convert into FUMP and FUTP in PRPS1 mutant Reh cells, suggesting a distinguishing mode of 5‐FU metabolism in ALL cells. To test this possibility, we next assessed 5‐FU metabolism in another ALL cell line Nalm‐6, as well as two colorectal carcinoma cell lines HCT116 and SW480. As shown in Figure 3E, both the ratios of 5‐FU converted to FdUMP in Reh and Nalm‐6 was significantly lower than that in HCT116 and SW480, though the outputs of FUMP and FUTP were still abounded in four cell lines.

### 3.4 OPRT is required for the chemosensitivity of ALL cells to 5‐FU

**Figure 4:**
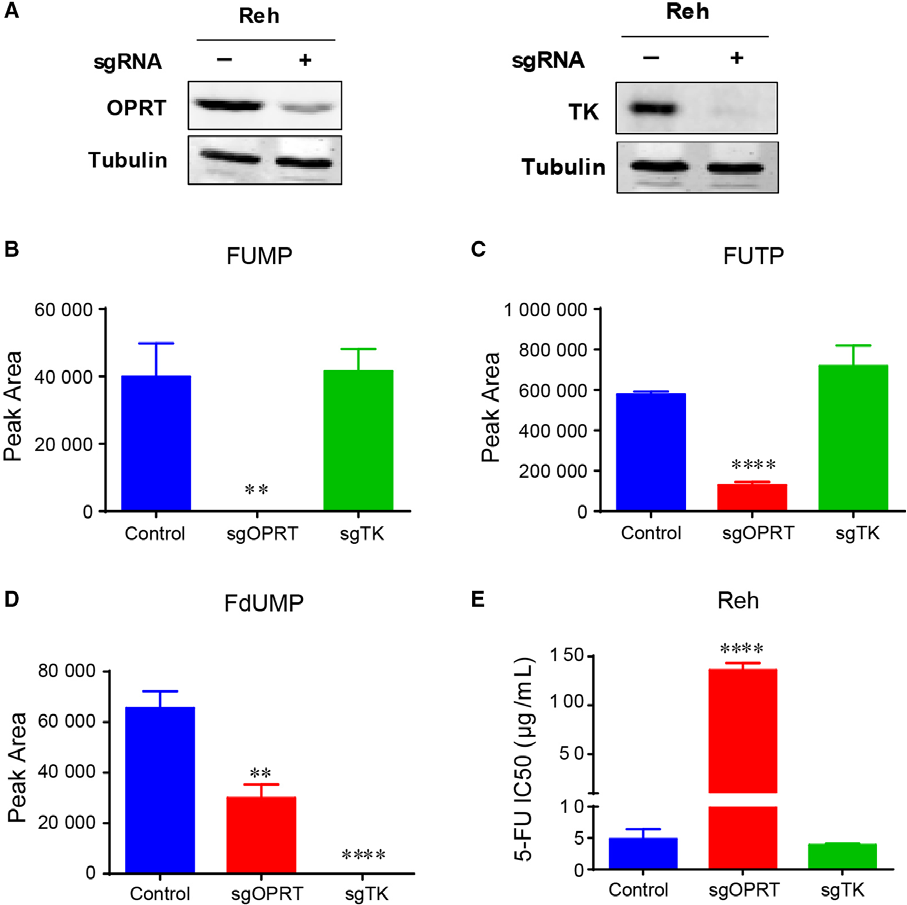
OPRT is required for the chemosensitivity of ALL cells to 5‐FU. (A) OPRT and TK in Ren cells were knocked down by using CRISPR‐Cas9 technology, respectively. (B–D) Conversion of 5‐FU to FUMP, FUTP and FdUMP in Reh cells when OPRT or TK was knocked down. **P < 0.01, ****P < 0.0001, two‐tailed Student's *t* tests. (E) Knocking down OPRT caused a dramatic resistance of Reh cells to 5‐FU analyzed by measuring IC50. ****P < 0.0001, two‐tailed Student's *t* tests

To further confirm the action of 5‐FU on ALL cells, we knocked down OPRT and TK in Reh cells by using CRISPR‐Cas9 technology (Figure 4A, Figure S2A and B). Both of OPRT and TK is essential for 5‐FU metabolism (Figure 3A), and they are required for the catalytic production of FUMP (or FUTP) and FdUMP, respectively (Figure 4B‐D). Then, we assessed the viability of Reh cells in response to 5‐FU by measuring IC50 when OPRT and TK knocked down. As shown in Figure 4E, knocking down OPRT caused a dramatic resistance to 5‐FU (almost 30 fold), while knocking down TK showed little effect on 5‐FU treatment. Taken together, these results indicated that 5‐FU sensitizes PRPS1 ALL cells by promoting its conversion to FUMP and FUTP.

### 3.5 Accumulated PRPP enhances sensitivity of PRPS1 mutant ALL cells to 5‐FU

**Figure 5:**
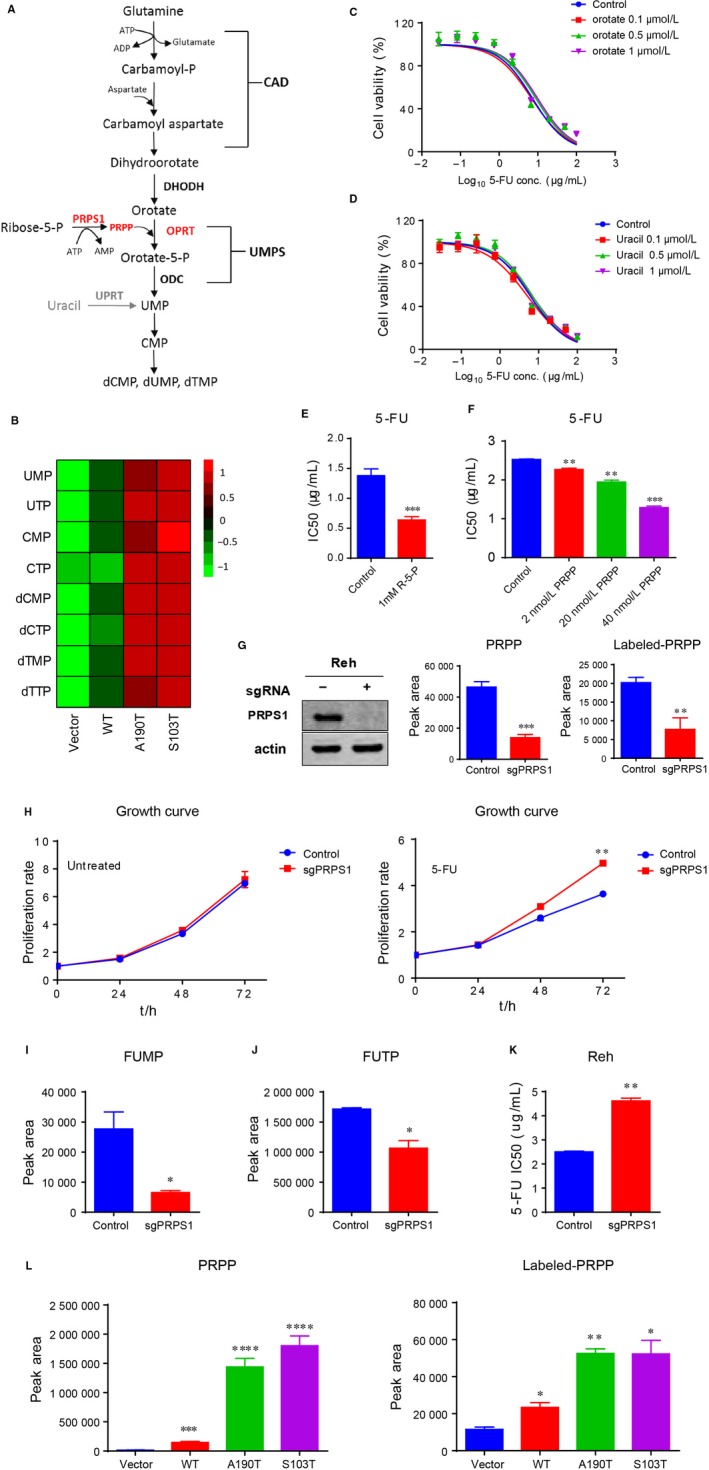
Accumulated PRPP enhances chemosensitivity of PRPS1 mutant ALL cells to 5‐FU. (A) Schematic depicting pyrimidine biosynthesis. (B) Pyrimidine nucleotides were increased in PRPS1 mutant cells compared to PRPS1 WT or control cells as showed by Heatmap. (C and D) Extra orotate and uracil could not sensitize Reh ALL cells to 5‐FU. (E and F) Extra R‐5‐P or PRPP enhanced Reh cell sensitivity to 5‐FU. **P < 0.01, ***P < 0.001, two‐tailed Student's *t* tests. (G) Knocking down PRPS1 by using CRISPR‐Cas9 technology reduced the levels of intracellular PRPP in Reh cells. PRPP was detected by LC‐MS, **P < 0.01, ***P < 0.001, two‐tailed Student's *t* tests. (H) PRPS1 knockdown cells proliferated much faster than control cells with the treatment of 5‐FU. Cell proliferation was measured at 0, 24, 48 and 72 hours after adding 1.5 μg/mL 5‐FU. **P < 0.01, two‐way ANOVA (multiple comparison‐analysis of different treatment groups at the same time). (I and J) Knocking down PRPS1 resulted in impaired conversion of 5‐FU to FUMP and FUTP in Reh cells. *P < 0.05, two‐tailed Student's *t* tests. (K) Knocking down PRPS1 caused a significant resistance of Reh cells to 5‐FU. **P < 0.01, two‐tailed Student's *t* tests. (L) Intracellular PRPP was significantly accumulated in Reh cells harboring PRPS1 mutants compared to control cells. *P < 0.05, **P < 0.01, ***P < 0.001, ****P < 0.0001, two‐tailed Student's *t* tests

PRPS1, as a rate‐limiting purine biosynthesis enzyme, is also essential for de novo synthesis of pyrimidine nucleotides (Figure 5A). Indeed, we observed much higher abundance of pyrimidine nucleotides in Reh cells harboring PRPS1 mutants compared to the cells harboring PRPS1 WT and control cells (Figure 5B). 5‐FU can convert to FUMP via normal pyrimidine biosynthesis by replacing orotate and uracil, we therefore investigated the effect of orotate and uracil on the chemosensitivity of ALL cells to 5‐FU. As shown in Figure 5C and D, there was no substantial change on Reh cell viability whether or not the cells were pretreated with orotate and/or uracil, indicating no competitive inhibition between 5‐FU and orotate/uracil. PRPS1 directly catalyzes ribose‐5‐phosphate (R‐5‐P) to produce PPRP, which is the important cofactor required for direct conversion of 5‐FU to FUMP by OPRT. We therefore assessed the role of PRPP in Reh cell sensitivity to 5‐FU. As shown in Figure 5E and F, extra adding of R‐5‐P or PRPP in culture medium could enhance Reh cell sensitivity to 5‐FU. Otherwise, the proliferation rate of Reh cells was significantly decreased with the treatment of R‐5‐P or PRPP (Figure S2D and E). We further knocked down PRPS1 to reduce the levels of intracellular PRPP in Reh cells (Figure 5G and Figure S2C). The data from cell proliferation assay showed that knocking down PRPS1 did not affect the proliferation of Reh cells under normal circumstances (Figure 5H, the left), whereas the PRPS1 knockdown cells proliferated much faster than control cells with the treatment of 5‐FU (Figure 5H, the right). We next showed that knocking down PRPS1 resulted in impaired conversion of 5‐FU to FUMP and FUTP in Reh cells (Figure 5I and J), and consequently, a significant resistance of Reh cells to 5‐FU (Figure 5K), indicating that PRPP reduction can reduce the sensitivity of Reh cells to 5‐FU. To provide insight into how PRPS1 mutants (A190T and S103T) caused the chemosensitivity of ALL cells to 5‐FU, we thus examined the production of PRPP in PRPS1 mutant Reh cells. As shown in Figure 5L, intracellular PRPP was significantly accumulated in Reh cells harboring PRPS1 mutants compared to control cells. Altogether, these results supported the conclusion that increased intracellular PRPP promotes 5‐FU prodrug activation and confers much more sensitivity to 5‐FU on PRPS1 mutant ALL cells.

## 4 DISCUSSION

Uncontrolled cell proliferation, as one of the characteristics of tumor cells, requires abundant nucleotides to support DNA and RNA synthesis.^17^ PRPS1, a rate‐limiting enzyme for nucleotide biosynthesis, plays an important role in maintaining intracellular nucleotide level and cell growth.^18^ Recent studies showed that expression level of PRPS1 is critical for tumorigenesis and clinical prognosis of cancer patients, based on the findings that both mRNA level and protein level of PRPS1 are up‐regulated in human colorectal cancer samples, and patients with high PRPS1 expression show poor prognosis.^19^ Furthermore, reduced expression of PRPS1 impairs tumor cell proliferation. In contrast, PRPS1 also remarkably affects cell proliferation and colony formation in glioblastoma stem cells.^20^ We previously identified relapse‐specific *PRPS1* mutations in childhood B‐ALL samples, and uncovered that ALL individuals who harbored *PRPS1* mutations relapsed early during treatment. Mechanistic studies further revealed that drug‐resistant PRPS1 mutants caused increased purine nucleotide biosynthesis by a mechanism of gain‐of‐function, and thus, elevated hypoxanthine competitively inhibits thiopurine conversion, conferring resistance to thiopurines.^5^ In despite of that, lometrexol, an inhibitor of *de novo* purine synthesis, can abrogate PRPS1 mutant‐driven thiopurine resistance.^5^ However, no chemotherapeutic drug has been shown to directly target PRPS1 mutant cells and thereby overcome relapse‐specific PRPS1 mutant‐conferred drug resistance. Here, we demonstrated that 5‐FU can target PRPS1 mutant Reh cells, causing enhanced DNA damage response and accelerated apoptosis.

5‐FU, an antimetabolite drug widely used in the treatment of several cancers including colorectal and breast cancers, exerts its anticancer effects through inhibition of TS and incorporation of its metabolites (FdUMP, FUTP, and FdUTP) into RNA and DNA.^21^, ^22^, ^23^ On the other hand, TS could also be used to predict the sensitivity of tumor cells to 5‐FU as a molecular biomarker. For instance, co‐treatment with leucovorin (5′‐formyltertrahydrofolate), which can facilitate the binding of FdUMP to TS, was shown to increase toxicity of 5‐FU in many cancer cell lines.^7^, ^23^ PRPP, a catalytic product of PRPS1, is the precursor of nucleotide biosynthesis and the important cofactor required for the conversion of 5‐FU to FUMP by OPRT. It has been reported that MTX could promote the conversion 5‐FU to FUMP by inhibiting purine biosynthesis.^24^ However, whether PRPP has any role in 5‐FU resistance is still incompletely clear until now. Here, we showed that accumulation of intracellular PRPP, produced by consistently active relapse‐specific PRPS1 mutants, could promote 5‐FU prodrug activation and confers much more sensitivity to 5‐FU on PRPS1 mutant Reh cells, prompting that both the mutations in PRPS1 and the expression of PRPS1 are potential biomarkers that can be used to predict tumor sensitivity to 5‐FU.

## CONFLICT OF INTEREST

The authors declare no conflict of interest.
