# Supplementary figures and images for: Increase of PRPP enhances chemosensitivity of PRPS1 mutant acute lymphoblastic leukemia cells to 5‐Fluorouracil
Source: J Cell Mol Med. 2018 Sep 25;22(12):6202–12. doi: 10.1111/jcmm.13907 (PMC6237573; doi:10.1111/jcmm.13907)

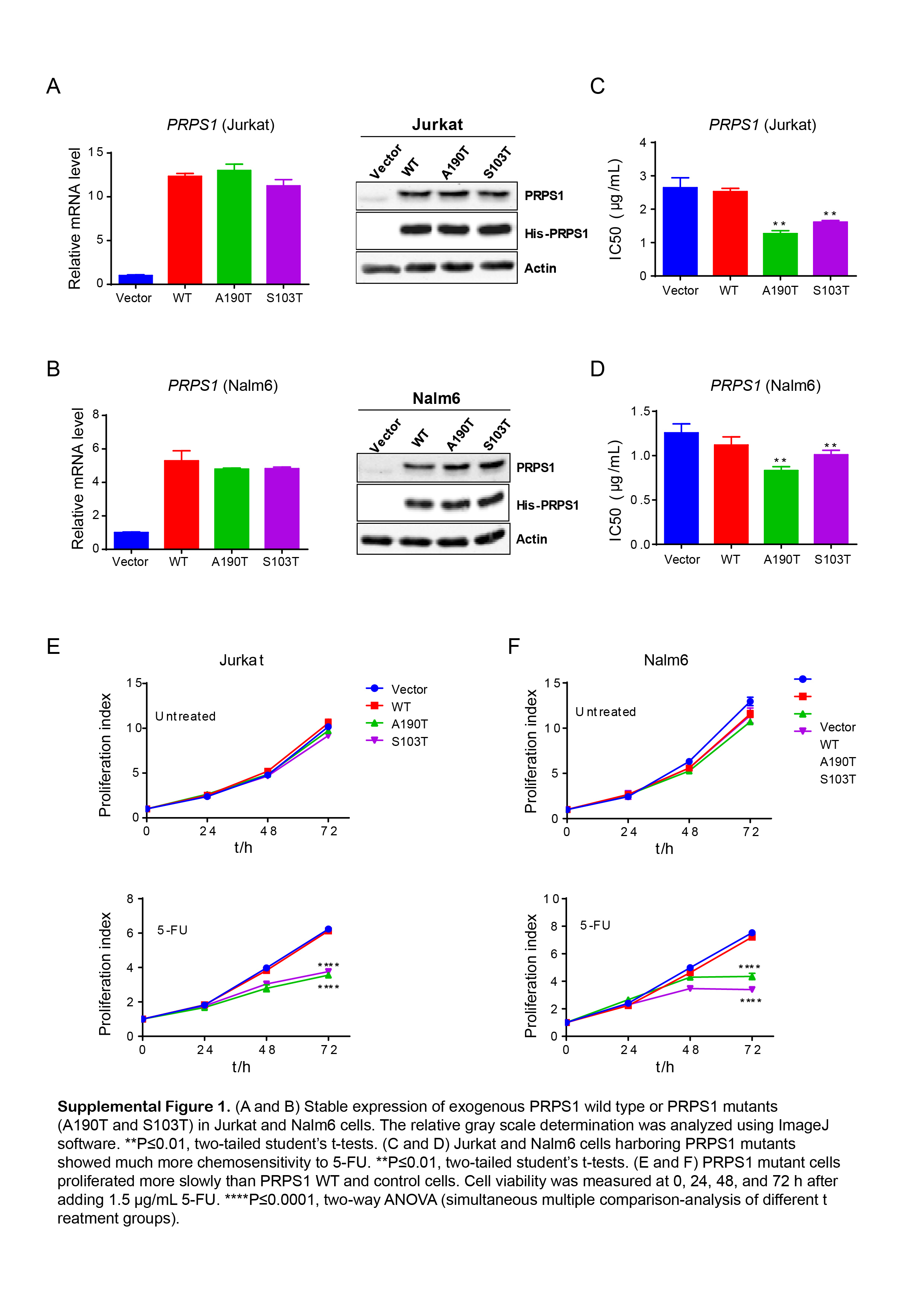

Supplement: Supplementary file 1 [file JCMM-22-6202-s001.jpg]

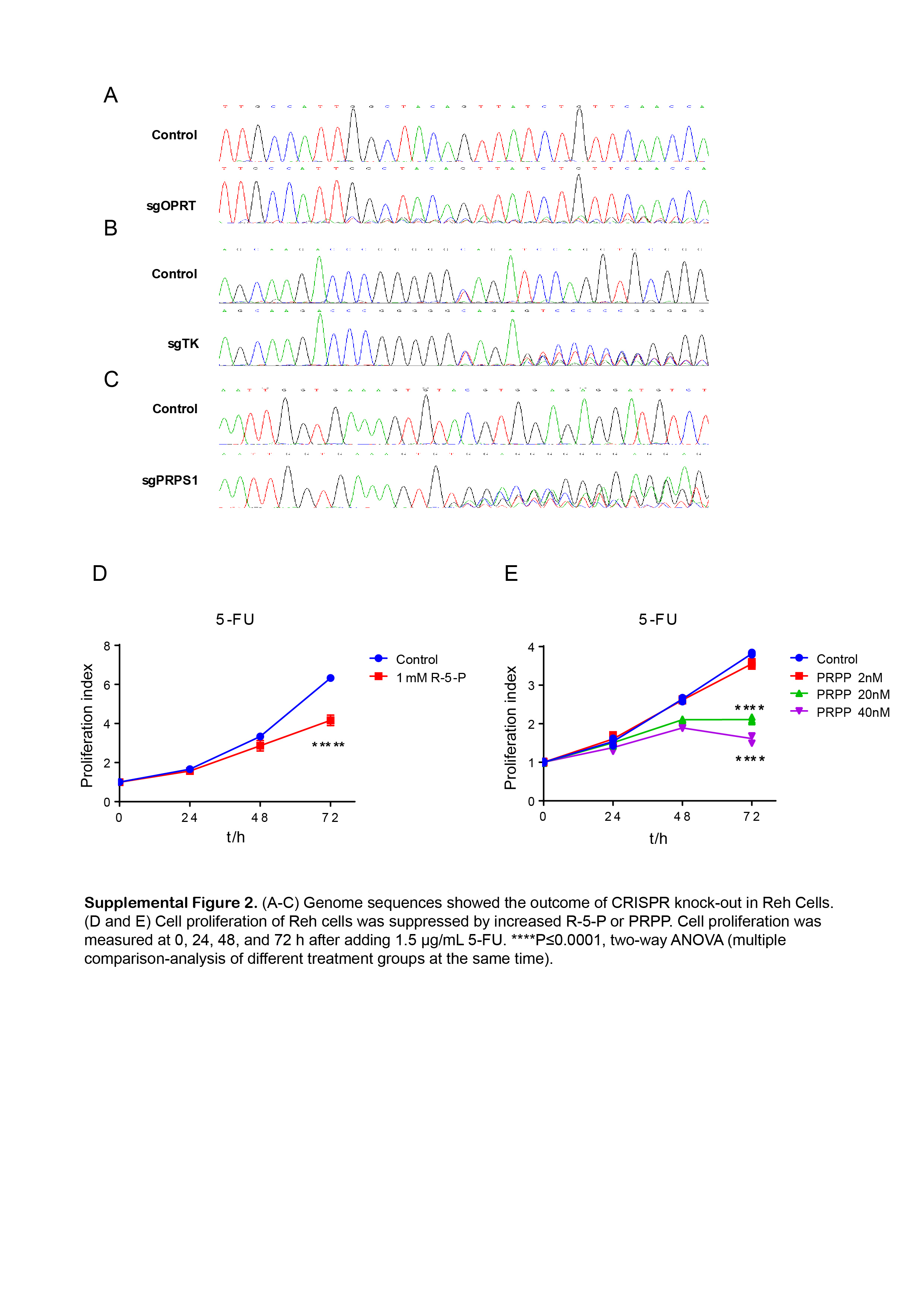

Supplement: Supplementary file 2 [file JCMM-22-6202-s002.jpg]
